# Finite Element Simulation of the Impedance Response of a Vascular Segment as a Function of Changes in Electrode Configuration

**DOI:** 10.2478/joeb-2020-0017

**Published:** 2020-12-31

**Authors:** M. Amini, H. Kalvøy, Ø.G. Martinsen

**Affiliations:** 1 Department of Physics, University of Oslo, Oslo, Norway; 2 Department of Clinical and Biomedical Engineering, Rikshospitalet, Oslo University Hospital, Oslo, Norway

**Keywords:** Bioimpedance, finite element simulation, electrode configuration

## Abstract

Monitoring a biological tissue as a three dimensional (3D) model is of high importance. Both the measurement technique and the measuring electrode play substantial roles in providing accurate 3D measurements. Bioimpedance spectroscopy has proven to be a noninvasive method providing the possibility of monitoring a 3D construct in a real time manner. On the other hand, advances in electrode fabrication has made it possible to use flexible electrodes with different configurations, which makes 3D measurements possible. However, designing an experimental measurement set-up for monitoring a 3D construct can be costly and time consuming and would require many tissue models. Finite element modeling methods provide a simple alternative for studying the performance of the electrode and the measurement set-up before starting with the experimental measurements. Therefore, in this study we employed the COMSOL Multiphysics finite element modeling method for simulating the effects of changing the electrode configuration on the impedance spectroscopy measurements of a venous segment. For this purpose, the simulations were performed for models with different electrode configurations. The simulation results provided us with the possibility of finding the optimal electrode configuration including the geometry, number and dimensions of the electrodes, which can be later employed in the experimental measurement set-up.

## Introduction

### Importance of monitoring biological tissues as a three dimensional model

Two dimensional (2D) cell cultures have been used as *in vitro* cell models for decades, however; a traditional two dimensional cell culture lacks the cell-cell communications in three dimensions as well as influential proteins that can be found only in a three dimensional (3D) extracellular matrix. In this way a 2D cell culture fails to mimic *in vivo* tissue complexity (1). Whereas, the cell-cell communication in a 3D environment in which the cells are both connected to each other and the extracellular matrix, forms a complex and dynamic system which adds a new dimension to the *in vitro* situations (2). 3D tissue models due to their close resemblance to an *in vivo* environment, make it possible to study the physiological responses of the cells in their native condition such as exchanging oxygen, nutrients and waste as well as monitoring their structural features (1, 3). Moreover, the 2D and 3D cell cultures differ not only quantitatively due to their dimensions but also qualitatively due to the cell behaviors such as: cellular morphology, proliferation rates, cell–extracellular matrix interactions (4), migration (5), gene expression, differentiation, signaling (6), physiological function, and electrophysiological properties (2, 7). Therefore, it is very important to monitor biological tissues as a three dimensional construct to be able to properly characterize their physiological and morphological properties and behaviors (7).

### Monitoring biological tissues using bioimpedance measurement techniques

A cell membrane acts as a very thin capacitor with high resistance. Therefore, when an alternating electrical signal is applied, the cell membrane acts as a capacitance. However, intracellular and extracellular fluids provide low resistivity (8,9,10). Consequently, the electrical impedance that the tissue produces under an alternating current is a complex quantity, which consists of a real and an imaginary part. The real part (R) is associated with the resistive pathways across the tissue such as intra and extracellular media. The imaginary part, the reactance (X), is associated with capacitive (C) and inductive (L) properties. Biological tissue and cells express capacitive current conduction through different structures as the cell membrane etc. that acts as capacitive pathways for alternating current. At the same time the inductive properties in biological tissues are typically negligibly small at frequencies below 10 MHz, when compared to their resistance and capacitance (11).

As the electrical impedance combines resistance and capacitance, it changes with variations in tissue anatomy, morphology, structure, and composition, as tissue components can demonstrate both conductive and charge storage properties.

Conductivity or current conduction in a biological tissue defines how well the tissue conducts electrical current. Electrical conductivity (σ) comprises of a non-frequency dependent component due to its ion content and their electric field induced mobility. Furthermore, a frequency dependent component associated with the energy absorbed by the dielectric relaxation process or dielectric loss factor, which defines the imaginary component of permittivity (12). On the other hand, permittivity (ɛ) defines the extent to which the tissue can be polarized under the applied electrical excitation. Relative permittivity (ɛ_r_) or dielectric constant is the ratio of the permittivity of a tissue to the permittivity of vacuum (13). Both electrical conductivity and relative permittivity vary widely between different biological tissues (14, 15).

The current conduction path in the tissue changes by altering the frequency of the applied signal. At low frequencies, cell membrane has a high resistance and the electric current can only travel through the extracellular fluid. However, at high frequencies, the reactance of the cell membrane decreases due to its capacitive behavior. At very high frequencies, the impedance of the membrane becomes very low and the current passes more uniformly through the tissue structure (11). Thus, measuring electrical impedance in different current conduction pathways by varying the signal frequency, makes it possible to analyze different tissue structures, composition and behaviors and study their anatomical, physiological and pathological status (9, 16).

Bioimpedance spectroscopy is a technique, which calculates and analyzes the electrical impedance from the boundary voltage/current measurements of the tissue at different frequencies. By employing bioimpedance spectroscopy not only the impedance of the tissue under study but also some other complex bioelectrical properties of the tissue such as dielectric relaxation and dispersions (*α*, *β*, and γ) (17) can be studied. Due to the ability of high frequency AC currents to penetrate the tissue, bioimpedance spectroscopy can be considered as a low cost, noninvasive and automation-compatible, label-free method to characterize the electrical properties of 3D tissue models in real time and with high temporal resolution (3).

### Influence of electrode configuration in bioimpedance spectroscopy measurements

Proper electrode configuration is substantial in establishing an optimized measurement set-up with precise performance, and includes electrode geometry, layout, dimension, structure and material (1, 3). Due to the importance of the dimensions of the electrodes, in some studies, theoretical calculations are performed to design the shape and size of the electrodes, prior to the experimental measurements (3, 18,19,20).

As the electrodes would be in close contact with the tissue under measurement as well as the culturing medium, it is very important that the electrode material would be chosen of a chemically and physically stable material that would not cause any kind of toxicity during the experiment. Some of the commonly used materials with nontoxic properties for this purpose are gold, platinum, palladium, and titanium (21,22,23,24). The other material, which is commonly used due to its low cost and stable potential is silver chloride (25). However, long term contact with biological tissue and culturing medium can cause erosion of the material and loss of silver chloride coating which lead to cytotoxicity due to change in electrode potential (22, 25, 26). So even by using stabilizing coatings, the silver chloride electrodes are less biocompatible than the electrodes made of gold, platinum and titanium (3).

Optimization of measurement performance by changing the electrode configuration and material has been done by many researchers before. Fosdick and Anderson (27) used the amperometric response of a flow detector for optimization of the design of its microelectrode arrays. Min and Baeumner (28) studied optimization of oxidation and reduction reactions of potassium ferro/ferrihexacyanide by changing the geometry and material of the interdigitated ultra-microelectrode arrays in a measurement system with a two electrode set-up. Sandison *et al.* (29) showed the dependence of the electrochemical behavior of micro-electrode arrays on the center-to-center spacing of the arrays. Lempka and coworkers (30) studied optimization of the signal to noise ratio and capability of silicon based microelectrode arrays for recording neural activities as a function of changing the electrode contact size. Wang and colleagues (31) investigated variations in the sensitivity of interdigitated microelectrode arrays as the result of changes in electrode dimension as well as frequency for electric cell-substrate impedance sensing (1).

A 3D measurement set-up should employ an electrode configuration, which would provide monitoring of the electrical signal propagation in the tissue construct in a three-dimensional manner. Performance of a 3D monitoring electrode is not only dependent on the electrode layout including the shape and size, and arrangement of the electrodes and their material, but also the curvature of the sensing area. A flexible electrode substrate provides the possibility of performing 3D measurements of the tissue while this is not possible with a conventional 2D electrode platform (7).

In order to achieve a solid integration between the electrodes and the 3D tissue, both the electrodes’ arrangement and dimensions as well as the mechanical properties of the electrode and its substrate should be compatible with the tissue to be monitored (32). Some researchers have reported using flexible and/or stretchable electrodes and electrode substrates, which due to their flexibility conform to the surface of the tissue to be monitored (33,34,35,36,37). Some of these flexible electrodes have been used in monitoring the electrical activities near surfaces of the heart (33,34,35), brain (37) and skin (36).

### The role of simulation and defining a finite element model

Designing the optimal measurement set-up requires performing the experiment several times and with various set-ups, which would be costly and time-consuming (38). Simulation using Finite Element Modeling (FEM) is a tool, which would facilitate understanding the tissue behavior and electrical response by visualization of current conduction paths in different parts of the model as well as the possibility of analyzing the contribution of each part of the model to the measurement results from the whole model. It also demonstrates the dependency of impedance and its real and imaginary components on the cell compositions such as cell membrane and intra and extracellular fluids (16). COMSOL Multiphysics is one of the simulation softwares that can demonstrate current conduction paths through 3D tissue models in multi-frequency bioimpedance measurements and in this way be employed as a practical tool in finding the optimized measurement set-up for monitoring 3D tissue models (1).

In a simulation approach for studying the electrical response of a model, the concept of impedance network can be used (39), where the model under simulation is divided into its components, where each of those components have their specific electrical properties such as electrical conductivity, relative permittivity and current density, etc. (40). Then the model components would be polarized using an electric field and then numerical calculation of impedance, capacitance and all other related electrical properties of the model components would be performed (41).

As in impedance measurement set-ups usually small dimensions (mm) and low frequencies (Hz to MHz) are used, therefore, the electrical field wavelength is much larger than the dimensions of the simulated sample and thus in simulating such measurement set-ups, a quasi-static approximation would typically be used (42, 43).

## Aim of the study

This study has been designed to develop a tool for optimizing a measurement set-up in which a flexible electrode is employed to facilitate 3D monitoring of a venous segment and provide measurements of high precision and accuracy. Therefore, the aim of the current study is employing finite element methods for simulating different measurement set-ups through building models with different electrode configurations (electrode number and dimensions). In addition, the finite element simulation would provide information about the contribution of each part of the model to the impedance measured from the whole system. We would expect that the optimized measurement set-up is obtained in the model in which venous segment has the highest contribution to the total measured impedance.

## Methods

The AC/DC module of the COMSOL Multiphysics® platform version 5.4 (COMSOL Inc., Burlington, MA, USA), was employed to perform the finite element simulations for different frequencies (44).

### Defining the model geometry and assigning material properties

**Figure 1:**
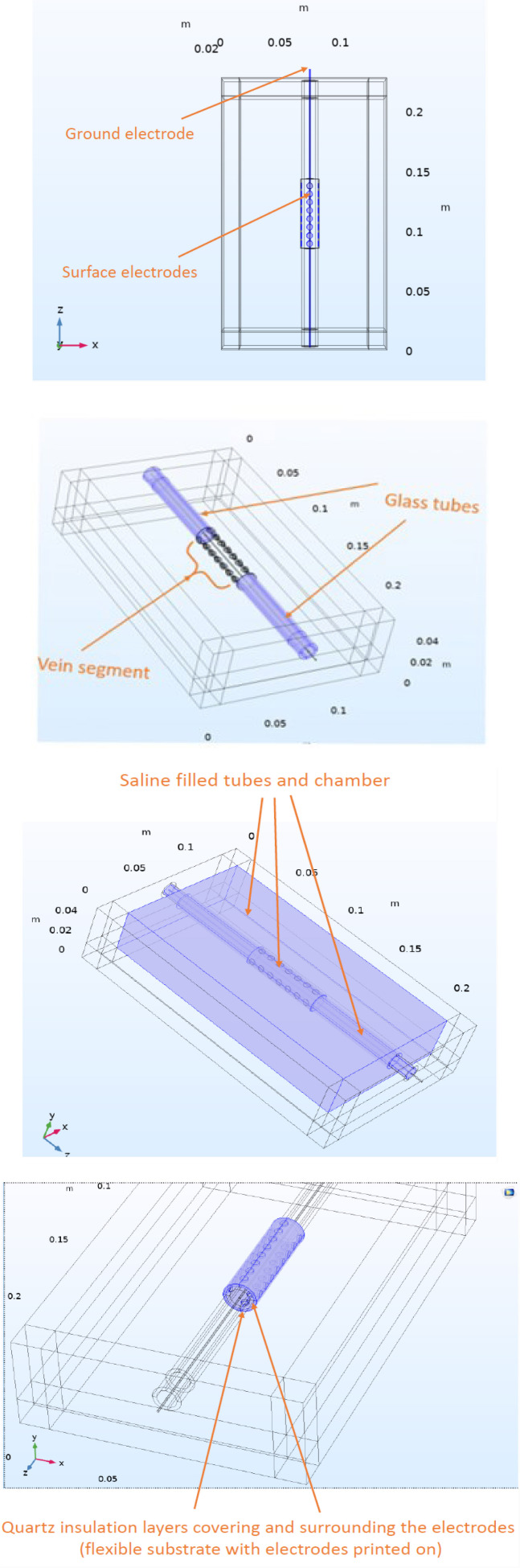
Different constituents of the COMSOL 3D finite element model.

The 3D model (figure 1) consists of a chamber out of PMMA or Poly methyl methacrylate also known as acrylic or Plexiglas, which is filled with saline solution and two glass tubes (silica) which are mounted in the chamber by passing through the holes fixed in the walls of the chamber. A venous segment in a cylindrical form with the length of 6 cm and diameter of 1.4 cm is mounted between these two glass tubes and the whole tube construct including glass tubes and the vein segment is filled with saline. The venous segment is surrounded by a thin layer of isolating material (quartz) as the flexible electrode substrate, which is rolled around the tissue in a cuff form.

Tiny golden cylinders, which are placed on the inner surface of the cuff towards and in contact with the venous segment are considered the surface electrodes, whereas the golden cylindrical bar passing through the vein and glass tubes is considered the ground electrode in this model.

Various numbers of surface electrodes (28, 32, 44 and 48) as well as different dimensions for both type of electrodes (r_terminal_ = 1, 2, 2.5 mm and r_ground_ = 0.5, 1 mm) were chosen. These were chosen the way that it would practically conform to the experimental set-up to be used later for measurements on a venous segment with the abovementioned dimensions. Gold was chosen as the electrode material in this model due to its high electrical conductivity, biocompatibility, and low electrode polarization (21,22,23,24).

The simulation system is defined by the dimensions and dielectric properties of the different components of the model (43). Due to the dependency of impedance measurements to permittivity and conductivity, the electrical properties used were frequency dependent electrical conductivity and relative permittivity (17). Therefore, the values for the electrical conductivity and relative permittivity of the different parts of the model were obtained from the inbuilt dielectric property tables for different materials in the COMSOL Multiphysics library in the way that they would match the values of the materials used for the experimental measurements later (38, 45).

### Defining the electrodes and electrical sources

A two-electrode configuration model with a voltage difference of 1 V was chosen. The same terminal feature was used for all the tiny surface golden electrodes were the voltage was applied, while the wire passing through the glass tubes and the venous segment was chosen as the ground electrode (figure 1) and the current flowing from the terminal electrodes to the ground electrode was calculated. Through Ohm's law, admittance (Y) was obtained and then the impedance (Z) was calculated as its inverse (Z = 1/Y). The impedance calculated this way is the impedance measured between the terminal and the ground electrode.

For our model, two different simulation methods were chosen and performed. In the first method, all the surface electrodes were considered as the terminal electrodes and the excitation was applied to all of them at the same time. While in the second method, one surface electrode at the time was chosen as terminal electrode to which the voltage was applied by turn. In this way, each surface electrode was employed as the terminal once during the simulation process.

### Meshing

**Figure 2:**
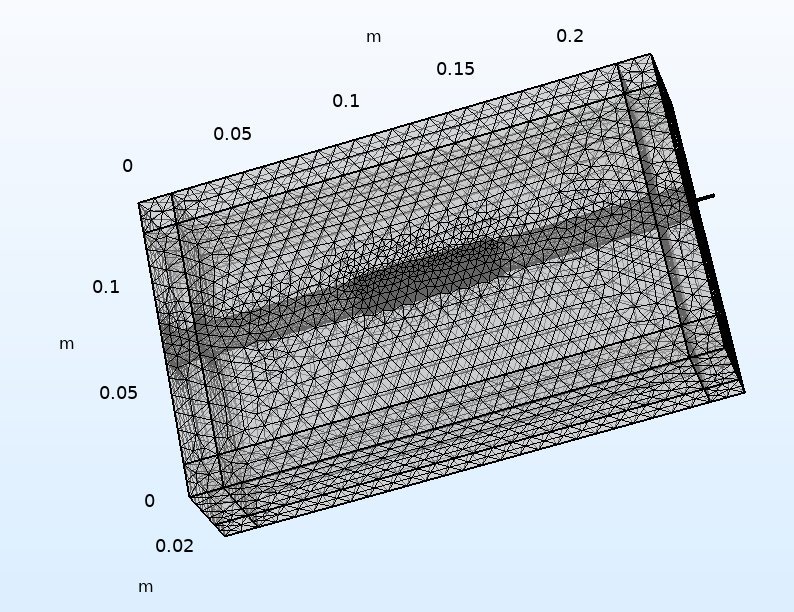
Extra fine mesh created for the model

In this stage, an extra fine mesh (figure 2) was created. This mesh was then merged with electrical properties of the material chosen to create the model (43). Meshing is done for subdividing the model into non-overlapping elements in the three dimensional space. The response of the whole model is the result of assembly of all the elements. Mesh has different levels from coarse to extra fine. If a fine mesh is chosen, the accuracy of the model would be increased. For example, a finer mesh provides a better representation of curved edges due to the smaller elements. However, this way the simulation tool has more data to calculate, therefore more time and memory is needed (38).

### Solving by using the frequency-dependent study type

Since the wavelength of the electric field in the different layers is much larger than the dimensions of the electrodes, a quasi-static electrical conduction model was applied to solve for the electric field in the saline solution (15, 44). The finite element simulation using the 3D AC/DC module of COMSOL Multiphysics was then performed for impedance measurements in the model. After obtaining the solution for the electric potential, boundary integration was used to determine electric current and consequently obtaining the electric impedance. With the AC/DC module of COMSOL it is possible to obtain the solution for the electric impedance at different frequencies. Simulations were repeated for frequencies exponentially distributed from 10 Hz to 1 MHz, to characterize the frequency response and the bioimpe-dance readings were recorded (46). The whole process was performed for different models with different number of surface electrodes (28, 32, 44 and 48) as well as different ground and terminal electrode dimensions (r_terminal_ = 1, 2, 2.5 mm and r_ground_ = 0.5, 1 mm).

Finally, to find the optimized electrode configuration for the measurement set-up, the contribution of the different parts of the model, especially the venous segment, to the impedance measured between the surface and the ground electrode (total impedance) was computed.

### Post processing

Some parameters that cannot be measured in a simple way can be studied by post processing the simulation results. These include electrical potential, current paths and current density, sensitivity, volume impedance density, volumetric energy loss density, real and imaginary parts of the impedance and consequently phase angle and impedance magnitude. Below some of these parameters are briefly explained.

#### Current density

Current density (J) is the electric current carried by a conductor per unit cross-sectional area of the conductor. Current density of every point throughout the measured area can be quantified by current density vectors (47). The current density vector is defined as a vector whose magnitude is the electric current per cross-sectional area at a given point in space, and its direction being that of the motion of the positive charges at this point (48). Current density is proportional to the electric potential as expressed by:

[1] J=σE

where *J* is the current density vector, *E* is the electric potential and *σ* is the electrical conductivity.

#### Sensitivity and volume impedance density

Sensitivity (S) is the ratio of the differential change in impedance to the differential change in conductivity within a voxel (49). The point sensitivity of impedance indicates how much the resistivity of the particular point influences the total impedance. Sensitivity is given by the following equation for a two-electrode system (50):

[2] S=Jec2I2

where *J_ec_* is the current density due to current passed through the model and *I* is the magnitude of the current, assumed constant through both the terminal and ground electrodes (38).

If the sensitivity (*S*) is multiplied with the complex resistivity (*ρ*) we get the volume impedance density (*z*) (38).

[3] z=ρS

The volume impedance density can be integrated over all points in the measured site to get the measured impedance, *Z* (38):

[4] Z=∫ ρS dV

Volume impedance density can be employed to find the contribution from each constituent of the model to the total impedance measured in the model (38).

#### Volumetric energy loss density

The complex admittance Y measured between the terminal and ground electrode (the inverse of the impedance) can be defined as:

[5] Y=G+iωC

where *G* is the conductance, *i* the imaginary unit, *ω* the angular frequency and *C* the capacitance (50). This applies if the measured material can be defined as a conductance, which is proportional to the dissipated part of the electric energy and a capacitance in parallel, which is proportional to the conserved part of the electric energy and with no induction (*L* = 0). This way, by integrating the total electric energy (conserved and dissipated) over different parts of the model, relative contribution of each part to the total capacitance and conductance and consequently the total calculated impedance, can be calculated (51).

In this model, volumetric energy loss density was computed as another simple way to find the contribution of different components of the model to the total measured impedance. This was done by integrating the power loss density over the components of interest and comparing with the overall power loss (over the whole model). It was assumed that due to the small phase angle, the dissipated energy was higher and dominant and was therefore chosen over the conserved energy.

#### Impedance magnitude and phase angle

Impedance is a complex parameter consisting of a real and an imaginary part. The real (Z_real_) and imaginary (Z_imag_) components of the lumped parameter of impedance (Z) which are called resistance (*R*) and reactance (*X*) respectively, can be computed by using the built-in functions and post processing methods in COMSOL simulation (43).

**Figure 3:**
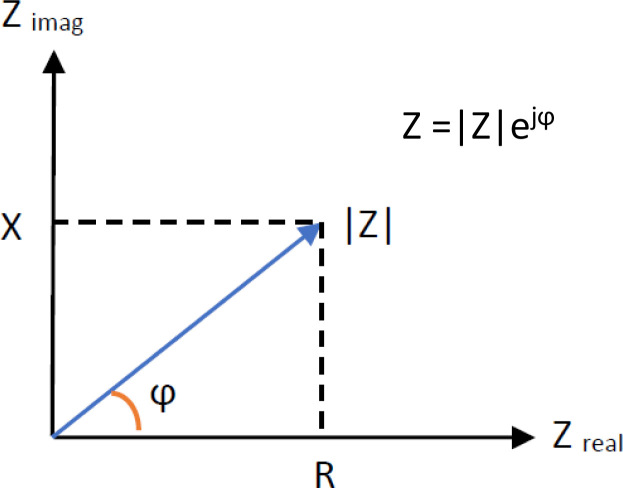
Diagram of the complex impedance illustrating the relationship between the real and imaginary parts of the impedance, where Z_real_ is the real or resistive component R of the impedance, whereas Z_imag_ is the imaginary or capacitive X component. |Z| and ϕ define the impedance magnitude and phase shift respectively.

Complex impedance can be described by polar coordinates (figure 3) (50). The impedance magnitude |*Z*| signifies the length of the impedance vector (3) and is defined as:

[6] $$\left|Z\right|=\sqrt{{\text{R}}^{2}+{\text{X}}^{2}}$$

The phase shift ϕ of the impedance describes the angle between the voltage and current (3) and can be defined as:

[7] $$\varphi =\arctan\;(\frac{X}{R})$$

Both impedance magnitude and the phase angle can be computed by post processing methods in FEM simulation.

### Ethical approval

The conducted research is not related to either human or animal use.

## Results

**Figure 4:**
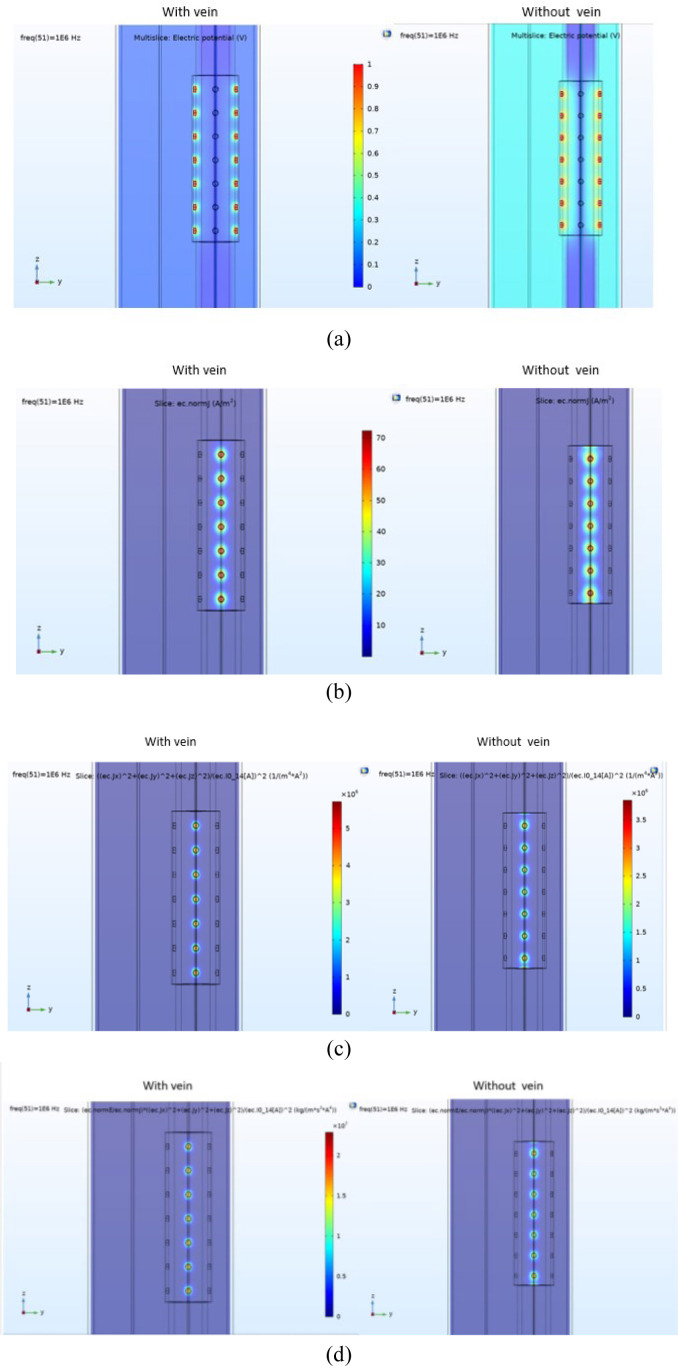
Y–Z Slice plots of a) electrical potential, b) current density, c) sensitivity and d) volume impedance density for the model with 28 surface electrodes simulated with and without the vein at the frequency of 1 MHz where all the surface electrodes were excited as terminals simultaneously (radius of the ground electrode = 0.5 mm, radius of the terminal electrodes = 1 mm).

**Figure 5:**
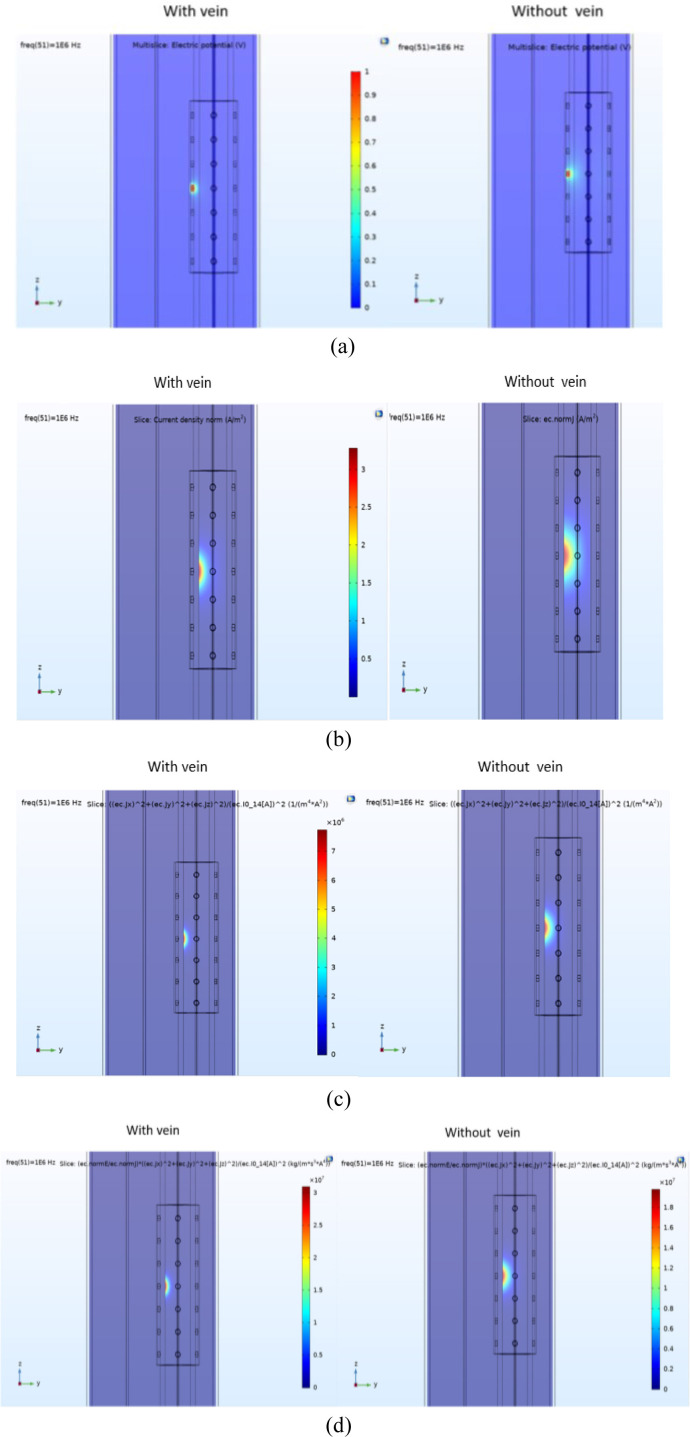
Y–Z Slice plots of a) electrical potential, b) current density, c) sensitivity and d) volume impedance density for the model with 28 surface electrodes simulated with and without the vein at the frequency of 1 MHz where only the middle electrode was excited as the terminal (radius of the ground electrode = 0.5 mm, radius of the terminal electrode = 1 mm).

Figures 4 and 5 demonstrate the results of the simulations performed in two ways. In figure 4 all the surface electrodes are considered as terminals and are excited at the same time, whereas in figure 5 only one surface electrode is employed and excited as the terminal.

In figures 4 and 5, parameters computed by post processing the simulation results, such as electrical potential, current density, sensitivity, and volume impedance density are demonstrated by using slice plots. These plots demonstrate the absolute values and non-vector parameters and do not provide any direction information (38). The simulations were performed both with and without the vein segment as shown in figures 4 and 5. In addition, some of these postprocessed parameters such as volume impedance density and volumetric power loss density were then employed to compute the contribution of different parts of the model to the impedance measurements in the whole model.

The simulation of the electrical response of the model visualized by the planar color plots demonstrates the effects of electrode configuration and geometry on measurement depth and sensitivity.

The detailed simulation results for different models as a function of changing the electrode configuration (number and dimension) are shown in 18 tables that due to their excessive numbers are placed in the appendix. Hereafter we refer to radius of the terminal and ground electrode as r_t_ and r_g_ consecutively. However, two electrode dimensions of (r_t_ = 1 mm and r_g_ = 0.5 mm) and (r_t_ = 1 mm and r_g_ = 1 mm) provided the highest impedance contribution from the vein for all number of surface electrodes. Therefore, the summary of the simulation results with these two electrode dimensions are shown in tables 1 and 2 where all the surface electrodes are employed as terminals and excited simultaneously.

**Table 1:** Simulations results including the percentage of the volumetric power loss density and volume integration of volume impedance density for vein and with the electrode dimensions of r_t_ = 1 mm and r_g_ = 0.5 mm where all the surface electrodes were excited simultaneously as terminals.

Number of electrodes	Volumetric power loss density in vein (%)	Volume impedance density for vein (%)
28	90.47%	90.48%
32	89.05%	89.06%
44	85.81%	85.82%
48	85.51%	85.59%

**Table 2:** Simulation results including the percentage of the volumetric power loss density and volume integration of volume impedance density for vein and with the electrode dimensions of r_t_ = 1 mm and r_g_ = 1 mm where all the surface electrodes were excited simultaneously as terminals.

Number of electrodes	Volumetric power loss density in vein (%)	Volume impedance density for vein (%)
28	92.80%	92.81%
32	91.48%	91.48%
44	89.28%	89.28%
48	89.06%	89.06%

In table 3, the results of two different simulations are compared where both have employed only one surface electrode as the terminal but with different dimensions of (r_t_ = 1 mm and r_g_ = 0.5 mm) versus (r_t_ = 2.5 mm and r_g_ = 0.5 mm). The terminal electrode is employed each time in different positions (middle, first end closer to the ground electrode output, and second end) to see the influence of the location of the terminal electrode on the results and this is done regardless of the number of surface electrodes.

**Table 3:** Simulations results including the percentage of the volumetric power loss density and volume integration of volume impedance density for vein and with two different surface and ground electrode dimensions (r_t_ = 1 mm and r_g_ = 0.5 mm versus r_t_ = 2.5 mm and r_g_ = 0.5 mm), regardless of the number of surface electrodes (28, 32, 44 and 48) and with only one surface electrode excited as the terminal (middle electrode, electrode at the first end close to the ground electrode output or electrode at the second end)

Electrodes dimensions (mm)	Location of the terminal electrode	Volumetric power loss density in vein (%)	Volume impedance density for vein (%)
r_t_=1.0 mm r_g_= 0.5 mm	Middle	95.98%	95.98%
r_t_=1.0 mm r_g_= 0.5 mm	First end close to the ground electrode	95.97%	95.97%
r_t_=1.0 mm r_g_= 0.5 mm	Second end	95.96%	95.96%
r_t_=2.5 mm r_g_= 0.5 mm	Middle	89.93%	89.93%
r_t_=2.5 mm r_g_= 0.5 mm	First end close to the ground electrode	89.87%	89.87%
r_t_=2.5 mm r_g_= 0.5 mm	Second end	89.82%	89.82%

## Discussion

The 3D AC/DC – Quasi-statics module of COMSOL Multi-physics FEM version 5.4 was employed for simulating the electrode configuration and the measurement set-up designed for monitoring the impedance of a venous segment. The build-in graphics tool was used to draw the geometry of each component of the measurement setup to which material and relevant dielectric properties were then allocated. The geometry was meshed with extra fine density. The simulation was then performed to analyze impedance changes in a two-electrode configuration system by varying the number of the surface electrodes (28, 32, 44 and 48) and their dimensions (r_t_ = 1, 2, and 2.5 mm) as well as the dimension of the ground electrode (r_g_ = 0.5 and 1 mm). In this way, a complete set of simulations consisting of many models with different electrode configuration was performed, where the surface electrodes where either excited one by one or all simultaneously as the terminal electrodes.

### Terminal electrodes excited one by one

Our simulation results including the data in tables 1, 2 and 3 indicate that applying excitation to the surface electrodes one by one as the terminal provides higher contribution from the vein to the total measured impedance in comparison to when all the surface electrodes are employed as terminals and are excited at the same time.

Changing the dimensions of both the terminal and the ground electrodes influences impedance measurements. The results show that for the electrode dimensions of r_t_ = 1 mm and r_g_ = 0.5 and 1 mm, the impedance contribution of the vein to the total measured impedance is higher in comparison to that of other electrode dimensions. This is shown in table 3 where two different electrode dimensions (r_t_ = 1 mm and r_g_ = 0.5 mm) and (r_t_ = 2.5 mm and r_g_ = 0.5 mm) are compared while one surface electrode at a time is excited as the terminal electrode. This is regardless of the number of surface electrodes as increasing and decreasing the number of electrodes while keeping the dimension of the electrode constant, only moves the electrode slightly back and forth and the results have shown that the difference it makes in the measurements is so little that it can be neglected.

In addition, to investigate the influence of location for the surface electrode as the terminal, three different simulations were performed where one surface electrode at the first end closer to the ground electrode output, middle and the second end of the venous segment was chosen as the terminal electrode. The difference in contribution from the venous segments was found to be in the range of 0.1%, when comparing the different terminal electrode positions (Table 3). This shows that the terminal electrode position along the venous segment does not have a significant influence on the obtained sensitivity and that the results in figure 5c will be similar for all the terminal electrodes when they are excited one by one.

It can also be concluded from the results that the open space between the terminal electrodes is another important factor that should be chosen carefully to avoid blind spots along segment surface with too low sensitivity.

As for the ground electrode, according to our simulation results, increasing the radius of the ground electrode from 0.5 to 1 mm gave an increase to the contribution of the venous segment to some degree. However, it should be noted that the choices for the radius of the ground electrode would be limited considering the dimensions of the different venous segments that we are aiming for in our *ex-vivo* use of the method. The results suggest the choice of 1 mm for the radius of the ground electrode, however, the length of the ground electrode is more important. To obtain the same symmetry between the terminal and the ground electrode, for middle and end positions of the terminal electrode, we must have a ground electrode that is significantly longer than the whole length of the venous segment.

Exciting the surface electrodes as the terminal one at the time and by turn, requires a longer measurement time. However, each excited surface electrode provides impedance information specifically from the site proximal to the electrode. In this way, by combining the measurement results and electrode position, a high spatial resolution would be obtained. The signal to noise ratio will also be much higher for the single terminal measurement compared to simultaneous measurement with all terminals in parallel. Hence, a higher sensitivity to local changes in the venous segment will be feasible. This is especially useful when it comes to monitoring and studying the venous segment with possible local deformation or damages in its tissue layers and cellular structure, as the higher sensitivity and spatial resolution would make it easier to detect and localize damage on the venous segment.

### Terminal electrodes excited all simultaneously

Results from tables 1 and 2 show that when all the terminal electrodes are excited at the same time, for the electrode dimensions of r_t_ = 1 mm and r_g_ = 0.5 and 1 mm, the number of 28 and 32 surface electrodes provide higher impedance contribution for the vein.

By assuming that the superposition principle is valid for our electrode simulations, we can illustrate the sensitivity distribution over the complete venous segment area by simultaneous excitation on all the terminal electrodes (figure 4). With this method, we can locate possible blind spots between the electrodes where the sensitivity will be too low to detect local changes or damage in the venous segment. By investigating the sensitivity distribution as a function of terminal electrode size and position, we can optimize our electrode setup when it comes to size, number and geometrical distribution of our electrodes. Increasing the electrode spacing while keeping the same electrode dimensions causes a significant decrease in the maximum magnitude of the electric field, as the field becomes more spread (1). On the other hand, by increasing the number of electrodes we can obtain a more even current distribution and cover more of the area between the electrodes. In the simultaneous excitation setup, each electrode will contribute with an additional current path in parallel to all the other electrodes. All these current paths will contribute equally to the measured impedance, and the signal to noise ratio for local changes will decrease with the number of electrodes. Therefore, it can be expected that the sensitivity for each electrodes will be inversely proportional to the number of electrodes excited simultaneously. Thus, although applying excitation to all the surface electrodes at the same time decreases the measurement time, it also decreases the sensitivity and takes away the spatial resolution when it comes to detecting local structural changes or damages in the venous segment wall.

### General findings

In our simulations, the total impedance is obtained by multiplying sensitivity with the resistivity in each point and integrate over all points in the volume of interest. In practical use for monitoring the properties of venous segment, the sensitivity for the application typically will be defined as the capability to discover a change in the segment. Our results demonstrate that whatever configuration for the terminal electrodes excitation is chosen, improving sensitivity for the vein monitoring application is possible by changing the dimensions of both the terminal and ground electrodes. However, the dimension of the terminal electrode is way more determinant. Results for the measured impedance, volumetric power loss density and volume impedance density, indicate that for all electrode numbers employed in our simulations (28, 32, 44 and 48), the surface electrode with the radius of 1 mm and ground electrode with the radius of 1 mm provide the highest percentage of contribution to the measured impedance from the venous segment. The reason to this might be that terminal electrodes with smaller dimensions concentrate the current density more in the relatively thin venous wall, which is the site of interest rather than the saline suspension.

Improving the spatial resolution of the measurements is very much dependent on the configuration chosen for terminal electrode excitation. Aiming for detection of local changes in a venous segment, the one-by-one terminal excitation will provide a higher spatial resolution in comparison to the simultaneous excitation of all terminal electrodes.

For both terminal electrode excitation configurations, the spacing between the terminal electrodes plays an important role in determining current distribution in the venous segment wall and consequently the area to be monitored. Therefore, while designing the measurement set-up, the positioning of the electrodes should be optimized to avoid areas or sites between the electrodes with insufficiently low current density.

The results also suggest that volume impedance density is more useful than sensitivity in estimating the contributions of different model parts to the measured impedance (47). Moreover, it can be inferred from the computations of the percentage of volume impedance density and volumetric power loss density that these two factors provide almost the same value when it comes to studying the contribution of each part of the model to the total impedance measurements.

Excitation of the terminal electrodes one by one as the optimal configuration for the experimental set-up can be achieved by various solutions such as applying a multiplexer which can employ all the surface electrodes as the terminal electrode one by one during the measurement process. This would make it possible to perform a thorough scanning of the whole vein segment for the purpose of bioimpedance spectroscopy measurements.

## Conclusion

The finite element simulation method was used to evaluate the feasibility of using a two-electrode bioimpedance spectroscopy set-up to monitor a three-dimensional blood vessel model. Simulations showed that we, within the assumed tissue properties, could monitor the impedance in the model with sufficient focus on the venous segment. We also found that this simulation model would be a valuable tool to design and optimize an experimental set-up with sufficiently high sensitivity and spatial resolution. Hence, the proposed simulation model will reduce the time and expenses in our project by significantly reducing the use of *ex-vivo* animal models. Our project will be followed up with further optimization of the measurement set-up. Electrode configurations with an optimized combination of size, number and position of the terminal electrodes will be found by repeated simulations with emphasize on even current distribution and sensitivity focus in the vein segment.

## Appendix

**Table.1:** Impedance measurements at 1 MHz with all the 28 surface electrodes excited simultaneously as terminals (7 electrodes in 4 rows)

**Surface electrode radius (mm)**	**Ground electrode radius (mm)**	**Impedance between surface and ground electrode (Ω)**
2.5	0.5	1.2119E1 −1.1656E-2i
2.5	1.0	1.1050E1 −1.0267E-2i
2.0	0.5	1.5423E1 −1.5411E-2i
2.0	1.0	1.4601E1 −1.4506E-2i
1.0	0.5	3.2653E1 −3.4107E-2i
1.0	1.0	3.1826E1 −3.3228E-2i

**Table.2:** Impedance measurements at 1 MHz with one of the 28 surface electrodes as terminal (7 electrodes in 4 rows)

**Surface electrode radius (mm)**	**Ground electrode radius (mm)**	**Impedance between surface and ground electrode (Ω)**
2.5 (electrode at the end closer to the ground electrode)	0.5	2.6984E2 −3.3115E-1i
2.5 (electrode in the middle)	0.5	2.6967E2 −3.3335E-1i
2.5 (electrode at the other end)	0.5	2.7026E2 −3.4495E-1i

**Table.3:** Volumetric power loss density measurements at 1 MHz with all 28 surface electrodes excited simultaneously as terminals (7 electrodes in 4 rows)

**Surface electrode radius (mm)**	**Ground electrode radius (mm)**	**Power loss density in vein (W)**	**Power loss density in saline (W)**	**Power loss density in the whole model (W)**	**Power loss density in vein (%)**	**Power loss density in saline (%)**
2.5	0.5	3.1263E-2	9.9870E-3	4.1259E-2	75.77%	24.20%
2.5	1.0	3.6575E-2	8.6698E-3	4.5248E-2	80.83%	19.16%
2.0	0.5	2.6133E-2	6.2797E-3	3.2418E-2	80.61%	19.37%
2.0	1.0	2.9147E-2	5.0954E-3	3.4244E-2	85.11%	14.87%
1.0	0.5	1.3855E-2	1.4566E-3	1.5313E-2	90.47%	9.51%
1.0	1.0	1.4582E-2	1.1284E-3	1.5710E-2	92.80%	7.18%

**Table.4:** Volumetric power loss density measurements at 1 MHz with one of the 28 surface electrodes as terminal (7 electrodes in 4 rows)

**Surface electrode radius (mm)**	**Ground electrode radius (mm)**	**Power loss density in vein (W)**	**Power loss density in saline (W)**	**Power loss density in the whole model (W)**	**Power loss density in vein (%)**	**Power loss density in saline (%)**
2.5 (electrode at the end closer to the ground electrode)	0.5	1.6653E-3	1.8764E-4	1.8529E-3	89.87%	10.12%
2.5 (electrode in the middle)	0.5	1.6674E-3	1.8673E-4	1.8541E-3	89.93%	10.07%
2.5 (electrode at the other end)	0.5	1.6618E-3	1.8819E-4	1.8500E-3	89.82%	10.17%

**Table.5:** Volume integration of Volume Impedance Density (VID) measurements at 1 MHz with all the 28 surface electrodes excited simultaneously as terminals (7 electrodes in 4 rows)

**Surface electrode radius (mm)**	**Ground electrode radius (mm)**	**VID for vein (Ω)**	**VID for saline (Ω)**	**VID for vein + saline (Ω)**	**VID for the nonconductive parts (Ω)**	**VID for vein (%)**	**VID for saline (%)**
2.5	0.5	6.2527E-2 +1.4138E-5i	1.9974E-2 +1.4785E-5i	8.2501E-2 +2.8923E-5i	−6.4902E-5 +2.8425E-7i	75.78 %	24.21 %
2.5	1.0	7.3150E-2 +1.9518E-5i	1.7339E-2 +1.4010E-5i	9.0490E-2 +3.3528E-5i	−6.6240E-5 +4.2870E-7i	80.83 %	19.16 %
2.0	0.5	5.2266E-2 +1.3515E-5i	1.2559E-2 +1.0436E-5i	6.4826E-2 +2.3951E-5i	−5.2804E-5 +2.3455E-7i	80.62 %	19.37 %
2.0	1.0	5.8294E-2 +1.7023E-5i	1.0191E-2 +9.4960E-6i	6.8485E-2 +2.6519E-5i	−5.4264E-5 +3.4159E-7i	85.11 %	14.88 %
1.0	0.5	2.7710E-2 +9.7267E-6i	2.9132E-3 +2.7658E-6i	3.0623E-2 +1.2493E-5i	−2.5621E-5 +6.7807E-8i	90.48 %	9.51%
1.0	1.0	2.9163E-2 +1.0749E-5i	2.2568E-3 +2.3572E-6i	3.1420E-2 +1.3107E-5i	−2.6169E-5 +8.2649E-8i	92.81 %	7.18%

**Table.6:** Volume integration of Volume Impedance Density (VID) measurements at 1 MHz with one of the 28 surface electrodes as terminal (7 electrodes in 4 rows)

**Surface electrode radius (mm)**	**Ground electrode radius (mm)**	**VID for vein (Ω)**	**VID for saline (Ω)**	**VID for vein + saline (Ω)**	**VID for the nonconductive parts (Ω)**	**VID for vein (%)**	**VID for saline (%)**
2.5 (electrode at the end closer to the ground electrode)	0.5	3.3306E-3 +1.4766E-6i	3.7528E-4 +2.6723E-8i	3.7059E-3 +1.5034E-6i	−3.7961E-6 +5.9637E-9i	89.87%	10.12%
2.5 (electrode in the middle)	0.5	3.3347E-3 +1.4305E-6i	3.7346E-4 +7.4700E-8i	3.7082E-3 +1.5052E-6i	−3.8315E-6 +9.1808E-9i	89.93%	10.07%
2.5 (electrode at other end)	0.5	3.3236E-3 +1.5898E-6i	3.7638E-4 −8.8830E-8i	3.7000E-3 +1.5010E-6i	−3.9716E-6 +2.1288E-8i	89.82%	10.17%

**Table.7:** Impedance measurement at 1MHz with all the 32 surface electrodes excited simultaneously as terminals (8 electrodes in 4 rows)

**Surface electrode radius (mm)**	**Ground electrode radius (mm)**	**Impedance between surface and ground electrode (Ω)**
2.5	0.5	1.0653E1 −1.0485E-2i
2.5	1.0	9.8828E0 −9.6378E-3i
2.0	0.5	1.3421E1 −1.3592E-2i
2.0	1.0	1.2610E1 −1.2638E-2i
1.0	0.5	2.8212E1 −2.9215E-2i
1.0	1.0	2.7457E1 −2.8411E-2i

**Table.8:** Impedance measurement at 1MHz with one of the 32 surface electrodes as terminal (8 electrodes in 4 rows)

**Surface electrode radius (mm)**	**Ground electrode radius (mm)**	**Impedance between surface and ground electrode (Ω)**
1.0 (electrode at the end closer to the ground electrode)	0.5	8.4612E2 −9.3477E-1i
1.0 (electrode in the middle)	0.5	8.4727E2 −9.4202E-1i
1.0 (electrode at the other end)	0.5	8.4635E2 −9.4246E-1i

**Table.9:** Volumetric power loss density measurement at 1MHz with all the 32 surface electrodes excited simultaneously as terminals (8 electrodes in 4 rows)

**Surface electrode radius (mm)**	**Ground electrode radius (mm)**	**Power loss density in vein (W)**	**Power loss density in saline (W)**	**Power loss density in the whole model (W)**	**Power loss density in vein (%)**	**Power loss density in saline (%)**
2.5	0.5	3.4034E-2	1.2890E-2	4.6933E-2	72.51%	27.46%
2.5	1.0	3.9523E-2	1.1067E-2	5.0593E-2	78.11%	21.87%
2.0	0.5	2.9014E-2	8.2354E-3	3.7255E-2	77.87%	22.10%
2.0	1.0	3.2746E-2	6.9049E-3	3.9652E-2	82.58%	17.41%
1.0	0.5	1.5784E-2	1.9382E-3	1.7723E-2	89.05%	10.93%
1.0	1.0	1.6659E-2	1.5510E-3	1.8210E-2	91.48%	8.50%

**Table.10:** Volumetric power loss density measurement at 1MHz with one of the 32 surface electrodes as terminal (8 electrodes in 4 rows)

**Surface electrode radius (mm)**	**Ground electrode radius (mm)**	**Power loss density in vein (W)**	**Power loss density in saline (W)**	**Power loss density in the whole model (W)**	**Power loss density in vein (%)**	**Power loss density in saline (%)**
1.0 (electrode at the end closer to the ground electrode)	0.5	5.6717E-4	2.3768E-5	5.9093E-4	95.97%	4.02%
1.0 (electrode in the middle)	0.5	5.6644E-4	2.3691E-5	5.9013E-4	95.98%	4.01%
1.0 (electrode at the other end)	0.5	5.6695E-4	2.3827E-5	5.9077E-4	95.96%	4.03%

**Table.11:** Volume integration of Volume Impedance Density (VID) measurements at 1MHz with all the 32 surface electrodes excited simultaneously as terminals (8 electrodes in 4 rows)

**Surface electrode radius (mm)**	**Ground electrode radius (mm)**	**VID for vein (Ω)**	**VID for saline (Ω)**	**VID for vein + saline (Ω)**	**VID for the nonconductive parts (Ω)**	**VID for vein (%)**	**VID for saline (%)**
2.5	0.5	6.8068E-2 +1.1734E-5i	2.5775E-2 +1.9988E-5i	9.3843E-2 +3.1721E-5i	−6.7451E-5 +1.2849E-6i	72.53%	27.46%
2.5	1.0	7.9045E-2 +1.7764E-5i	2.2133E-2 +1.8676E-5i	1.0118E-1 +3.6440E-5i	−8.0453E-5 +1.0100E-6i	78.12%	21.87%
2.0	0.5	5.8027E-2 +1.2993E-5i	1.6467E-2 +1.3744E-5i	7.4495E-2 +2.6737E-5i	−6.2039E-5 +5.5682E-7i	77.89%	22.10%
2.0	1.0	6.5491E-2 +1.7554E-5i	1.3809E-2 +1.2381E-5i	7.9300E-2 +2.9935E-5i	−6.4510E-5 +6.7324E-7i	82.58%	17.41%
1.0	0.5	3.1567E-2 +1.0773E-5i	3.8756E-3 +3.4894E-6i	3.5443E-2 +1.4263E-5i	−2.9575E-5 +1.1871E-7i	89.06%	10.93%
1.0	1.0	3.3318E-2 +1.2025E-5i	3.1017E-3 +2.9791E-6i	3.6419E-2 +1.4999E-5i	−3.0186E-5 +1.3771E-7i	91.48%	8.51%

**Table.12:** Volume integration of Volume Impedance Density (VID) measurements at 1MHz with one of the 32 surface electrodes as terminal (8 electrodes in 4 rows)

**Surface electrode radius (mm)**	**Ground electrode radius (mm)**	**VID for vein (Ω)**	**VID for saline (Ω)**	**VID for vein + saline (Ω)**	**VID for the nonconductive parts (Ω)**	**VID for vein (%)**	**VID for saline (%)**
1.0 (electrode at the end closer to the ground electrode)	0.5	1.1343E-3 +4.9177E-7i	4.7536E-5 +1.5713E-8i	1.1819E-3 +5.0749E-7i	−1.0513E-6 +1.5073E-10i	95.97%	4.02%
1.0 (electrode in the middle)	0.5	1.1329E-3 +4.9362E-7i	4.7381E-5 +1.3227E-8i	1.1803E-3 +5.0685E-7i	−1.0588E-6 +7.3337E-10i	95.98%	4.01%
1.0 (electrode at the other end)	0.5	1.1339E-3 +5.0746E-7i	4.7651E-5	1.1815E-3 +5.0733E-7i	−1.0620E-6 +1.0793E-9i	95.96%	4.03%

**Table.13:** Impedance measurements at 1MHz with both all and one of the 44 surface electrodes as terminals (11 electrodes in 4 rows)

**Surface electrode radius (mm)**	**Ground electrode radius (mm)**	**Impedance between surface and ground electrode (Ω)**
1.0 (all electrodes)	1.0	2.1251E1 −2.2560E-2i
1.0 (all electrodes)	0.5	2.2121E1 −2.3553E-2i
1.0 (electrode at the end closer to the ground electrode)	0.5	8.4757E2 −9.3724E-1i
1.0 (middle electrode)	0.5	8.4507E2 −9.3706E-1i
1.0 (electrode at the other end)	0.5	8.4592E2 −9.4144E-1i

**Table.14:** Volumetric power loss density measurements at 1MHz with both all and one of the 44 surface electrodes as terminal (11 electrodes in 4 rows)

**Surface electrode radius (mm)**	**Ground electrode radius (mm)**	**Power loss density in vein (W)**	**Power loss density in saline (W)**	**Power loss density in the whole model (W)**	**Power loss density in vein (%)**	**Power loss density in saline (%)**
1.0 (all electrodes)	1.0	2.1008E-2	2.5198E-3	2.3529E-2	89.28%	10.71%
1.0 (all electrodes)	0.5	1.9396E-2	3.2042E-3	2.2603E-2	85.81%	14.17%
1.0 (electrode at the end closer to the ground electrode)	0.5	5.6628E-4	2.3644E-5	5.8992E-4	95.99%	4.01%
1.0 (electrode in the middle)	0.5	5.6795E-4	2.3717E-5	5.9167E-4	95.99%	4.01%
1.0 (electrode at the other end)	0.5	5.6727E-4	2.3802E-5	5.9107E-4	95.97%	4.02%

**Table.15:** Volume integration of Volume Impedance Density (VID) measurements at 1MHz with both all and one of the 44 surface electrodes as terminal (11 electrodes in 4 rows)

**Surface electrode radius (mm)**	**Ground electrode radius (mm)**	**VID for vein (Ω)**	**VID for saline (Ω)**	**VID for vein + saline (Ω)**	**VID for the nonconductive parts (Ω)**	**VID for vein (%)**	**VID for saline (%)**
1.0 (all electrodes)	1.0	4.2016E-2 +1.4290E-5i	5.0395E-3 +4.7016E-6i	4.7056E-2 +1.8991E-5i	−4.0449E-5 +3.4149E-7i	89.28%	10.71%
1.0 (all electrodes)	0.5	3.8792E-2 +1.2009E-5i	6.4084E-3 +5.6141E-6i	4.5200E-2 +1.7622E-5i	−3.9316E-5 +2.9663E-7i	85.82%	14.17%
1.0 (electrode at the end closer to the ground electrode)	0.5	1.1326E-3 +4.9125E-7i	4.7288E-5 +1.5428E-8i	1.1798E-3 +5.0668E-7i	−1.0507E-6 +1.6192E-10i	95.99%	4.01%
1.0 (one middle electrode)	0.5	1.1359E-3 +4.9613E-7i	4.7434E-5 +1.2071E-8i	1.1833E-3 +5.0820E-7i	−1.0576E-6 +7.5986E-10i	95.99%	4.01%
1.0 (electrode at the other end)	0.5	1.1345E-3 +5.0853E-7i	4.7603E-5 −9.1060E-10i	1.1821E-3 +5.0762E-7i	−1.0618E-6 +1.1214E-9i	95.97%	4.02%

**Table.16:** Impedance measurements at 1MHz with both all and one of the 48 surface electrodes as terminals (12 electrodes in 4 rows)

**Surface electrode radius (mm)**	**Ground electrode radius (mm)**	**Impedance between surface and ground electrode (Ω)**
1.0 (all electrodes)	1.0	1.9541E1 −1.8887E-2i
1.0 (all electrodes)	0.5	2.0357E1 −1.9872E-2i
1.0 (electrode at the end closer to the ground electrode)	0.5	8.4669E2 −9.6656E-1i
1.0 (middle electrode)	0.5	8.4367E2 −9.3392E-1i
1.0 (electrode at the other end)	0.5	8.5221E2 −1.0653E0i

**Table.17:** Volumetric power loss density measurements at 1MHz with both all and one of the 48 surface electrodes as terminal (12 electrodes in 4 rows)

**Surface electrode radius (mm)**	**Ground electrode radius (mm)**	**Power loss density in vein (W)**	**Power loss density in saline (W)**	**Power loss density in the whole model (W)**	**Power loss density in vein (%)**	**Power loss density in saline (%)**
1.0 (all electrodes)	1.0	2.2788E-2	2.7982E-3	2.5587E-2	89.06%	10.94%
1.0 (all electrodes)	0.5	2.1003E-2	3.5551E-3	2.4561E-2	85.51%	14.47%
1.0 (electrode at the end closer to the ground electrode)	0.5	5.6653E-4	2.4003E-5	5.9053E-4	95.93%	4.06%
1.0 (electrode in the middle)	0.5	5.6885E-4	2.3795E-5	5.9265E-4	95.98%	4.01%
1.0 (electrode at the other end)	0.5	5.6191E-4	2.4793E-5	5.8671E-4	95.77%	4.22%

**Table.18:** Volume integration of Volume Impedance Density (VID) measurements at 1MHz with both all and one of the 48 surface electrodes as terminal (12 electrodes in 4 rows)

**Surface electrode radius (mm)**	**Ground electrode radius (mm)**	**VID for vein (Ω)**	**VID for saline (Ω)**	**VID for vein + saline (Ω)**	**VID for the nonconductive parts (Ω)**	**VID for vein (%)**	**VID for saline (%)**
1.0 (all electrodes)	1.0	4.5575E-2 +1.6240E-5i	5.5963E-3 +4.3527E-6i	5.1171E-2 +2.0593E-5i	−3.6781E-5 +4.2610E-8i	89.06%	10.94%
1.0 (all electrodes)	0.5	4.1652E-2 +1.4285E-5i	7.0078E-3 +4.6332E-6i	4.8660E-2 +1.8918E-5i	−3.5480E-5 +2.6543E-8i	85.59%	14.40%
1.0 (electrode at the end closer to the ground electrode)	0.5	1.1331E-3 +5.2890E-7i	4.8006E-5 −2.1807E-8i	1.1811E-3 +5.0709E-7i	−1.0724E-6 +2.4808E-9i	95.93%	4.06%
1.0 (one middle electrode)	0.5	1.1377E-3 +4.9692E-7i	4.7590E-5 +1.2089E-8i	1.1853E-3 +5.0901E-7i	−1.0576E-6 +7.7056E-10i	95.98%	4.01%
1.0 (electrode at the other end)	0.5	1.1238E-3 +5.5811E-7i	4.9585E-5 −5.4704E-8i	1.1734E-3 +5.0341E-7i	−1.1555E-6 +8.2855E-9i	95.77%	4.22%
